# Discovering genes associated with dormancy in the monogonont rotifer *Brachionus plicatilis*

**DOI:** 10.1186/1471-2164-10-108

**Published:** 2009-03-13

**Authors:** Nadav Y Denekamp, Michael AS Thorne, Melody S Clark, Michael Kube, Richard Reinhardt, Esther Lubzens

**Affiliations:** 1 Israel Oceanographic and Limnological Research, Haifa 31080, Israel; 2 British Antarctic Survey, Natural Environment Research Council, High Cross, Madingley Road, Cambridge CB3 0ET, UK; 3 Max-Planck Insitute for Molecular Genomics, Berlin-Dahlem, Germany

## Abstract

**Background:**

Microscopic monogonont rotifers, including the euryhaline species *Brachionus plicatilis*, are typically found in water bodies where environmental factors restrict population growth to short periods lasting days or months. The survival of the population is ensured via the production of resting eggs that show a remarkable tolerance to unfavorable conditions and remain viable for decades. The aim of this study was to generate Expressed Sequence Tags (ESTs) for molecular characterisation of processes associated with the formation of resting eggs, their survival during dormancy and hatching.

**Results:**

Four normalized and four subtractive libraries were constructed to provide a resource for rotifer transcriptomics associated with resting-egg formation, storage and hatching. A total of 47,926 sequences were assembled into 18,000 putative transcripts and analyzed using both Blast and GO annotation. About 28–55% (depending on the library) of the clones produced significant matches against the Swissprot and Trembl databases. Genes known to be associated with desiccation tolerance during dormancy in other organisms were identified in the EST libraries. These included genes associated with antioxidant activity, low molecular weight heat shock proteins and Late Embryonic Abundant (LEA) proteins. Real-time PCR confirmed that LEA transcripts, small heat-shock proteins and some antioxidant genes were upregulated in resting eggs, therefore suggesting that desiccation tolerance is a characteristic feature of resting eggs even though they do not necessarily fully desiccate during dormancy. The role of trehalose in resting-egg formation and survival remains unclear since there was no significant difference between resting-egg producing females and amictic females in the expression of the *tps-1 *gene. In view of the absence of vitellogenin transcripts, matches to lipoprotein lipase proteins suggest that, similar to the situation in dipterans, these proteins may serve as the yolk proteins in rotifers.

**Conclusion:**

The 47,926 ESTs expand significantly the current sequence resource of *B. plicatilis*. It describes, for the first time, genes putatively associated with resting eggs and will serve as a database for future global expression experiments, particularly for the further identification of dormancy related genes.

## Background

The phylum Rotifera is a relatively small group of microscopic aquatic or semi-aquatic invertebrates, encompassing about 2,000 species of unsegmented, bilaterally symmetrical pseudocoelomates. The species under study, the monogonont rotifer *Brachionus plicatilis*, is a zooplanktonic invertebrate, typically found in water bodies where environmental factors restrict population growth to short periods lasting days or months. The adverse conditions for growth include evaporation of water in temporary habitats leading to desiccation, unfavorable temperatures, and lack of food or appearance of predators. This is a relatively hostile environment and the survival of the population in such conditions is ensured via the production of resting eggs. These show a remarkable tolerance to unfavorable conditions and may be stored for decades [1,2]. Their high reproductive rates facilitate colonization of vacant niches with extreme rapidity, converting primary production (algal and bacterial) into a form usable for secondary consumers with remarkable efficiency [3]. Consequently, the euryhaline rotifer *Brachionus plicatilis*, has been developed as an essential food source for raising marine fish larvae in marine fish hatcheries (reviewed in [4]).

**Figure 1:**
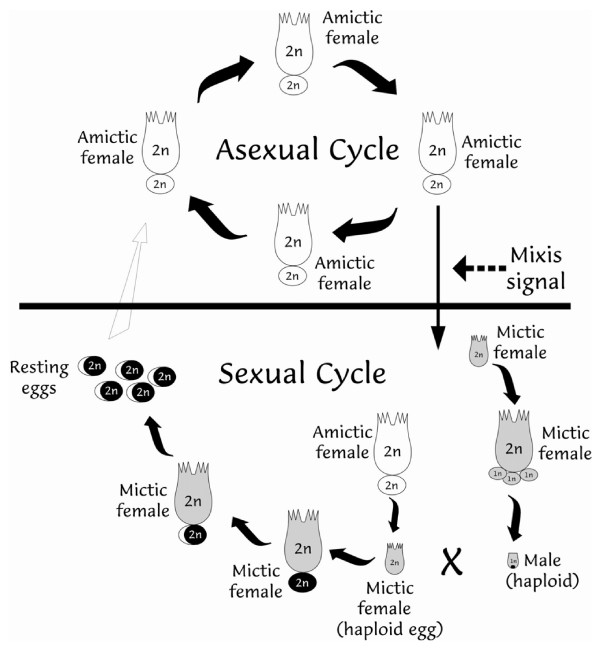
**The life cycle of *Brachionus plicatilis *showing asexual and sexual reproduction and formation of resting eggs**. In the asexual life cycle, diploid amictic females produce parthenogenetic diploid amictic eggs. A mixis signal initiates the occurrence of a sexual cycle, whereby, diploid mictic females produce haploid eggs via meiosis. The haploid eggs develop into either haploid males or, if fertilized, they form diploid dormant (or diapausing) resting eggs. The internal insemination of diploid mictic females carrying haploid eggs, is possible for only a few hours after birth. Mictic females are shaded in grey and include mictic females producing male eggs or mictic females that form diploid resting eggs. All females are diploid while males are haploid.

*Brachionus plicatilis *shows periodic parthenogenesis, where asexual reproduction is prevalent but under certain circumstances sexual reproduction occurs (Figure 1). Parthenogenesis dominates (amictic phase) the monogonont life cycle in the absence of males, but following certain environmental cues, sexual reproduction (mictic phase) takes place. Females that reproduce asexually are termed "amictic" and females that reproduce sexually are "mictic". Amictic females produce diploid eggs that develop by ameiotic pathenogenesis into females. Mictic females are morphologically similar to amictic females but produce haploid (mictic) eggs via meiosis. These eggs will develop parthenogenetically into haploid males but if these mictic females are fertilized they will produce diploid resting eggs. The haploid males are significantly smaller than the females and move faster. The mictic females produce resting eggs only if they are inseminated at a young age. Resting-egg production is therefore a consequence of switching from an asexual type of reproduction to sexual reproduction. Resting eggs then undergo obligatory diapause or dormancy, eventually hatching as amictic females [2,5-8]. It has also been suggested that certain clones show a higher tendency for sexual reproduction and resting-egg production than others, within the same population [9,10]. The factors inducing the mixis signal are largely unknown, although population density and environmental factors such as salinity, presence of pheromones and food availability have been shown to play a role [11-15].

Clues for the biological processes underlying the dormant stage can be inferred from other organisms, such as spores, plant seeds, cysts or resting eggs that display cryptobiosis where the metabolic rate is extremely low (~5% of normal) and associated with profound changes within the cell [16,17]. Cryptobiosis is associated with desiccation or anhydrobiosis and involves protectants that stabilize cellular macromolecules for periods extending many years [17]. These processes are often associated with the onset of stressful environmental conditions. However, production of rotifer resting eggs is not always stress related and resting eggs do not necessarily undergo desiccation during their dormant period and therefore it remains to be shown whether these eggs express genes associated with stress resistance and desiccation tolerance. To date, there have been very few molecular studies on *B. plicatilis *and these have been mainly limited to single gene studies on aging and environmental contamination [18-20] and a small-scale EST project [21]. None of these relate to resting-egg formation or dormancy.

The aim of the present study was to develop EST resources of *B. plicatilis *for future molecular analyses into the dormancy process in this organism. Four EST libraries were constructed: a mixed stage rotifer culture, females with resting eggs, resting eggs and resting eggs during hatching. The libraries were normalized in order to increase the chances of discovering rarely expressed genes. In addition, four subtractive libraries were constructed with the aim of enhancing the gene diversity of the ESTs. In this paper we characterize the EST libraries and identify putative genes associated with dormancy, formation and survival of rotifer resting eggs.

## Results and discussion

A total of 47,926 sequences remained after clone sequencing and quality checking and these had a minimum transcript size of 100 bp and an average of 538 bp. Sequencing effort was concentrated on the normalized libraries, which comprised 91.2% of the dataset, with between 21.5–24.9% contributed by each library (Table 1). Preliminary sequencing was performed on the subtractive libraries (approximately 1000 clones each) and therefore these only comprise 8.8% of the EST sequences (Table 2). The gene discovery and diversity rates of all libraries are relatively high (0.53–0.68 and 0.36–0.46 respectively) with the exception of the sbs04 subtractive library. Gene discovery is defined as the number of different "genes" each library contributed, divided by library size and gene diversity is defined as the number of singletons in each library divided by library size [22].

**Table 1:** General statistics for the normalized libraries.

	**MS**	**RE**	**REH**	**FRE**
# Reads*	11956	10659	10441	10340
**# average read length (bp)**	604	585	606	600
**# singletons**	4863	3881	4480	4205
**# clusters**	2380	1804	2014	2032
**# putative transcripts**	7243	5687	6494	6237
**Avg cluster size**	2.98	3.76	2.96	3.02
**Largest cluster**	13	31	19	18
**# clusters with 2 ESTs**	1251	854	1120	1106
**# clusters with 3 ESTs**	547	383	449	416
**# clusters with 4–5 ESTs**	428	294	304	349
**# clusters with 6–10 ESTs**	146	192	129	156
**# clusters with > 10 ESTs**	8	81	12	5
**Gene discovery**	0.61	0.53	0.62	0.60
**Gene diversity**	0.41	0.36	0.43	0.41
# (%) with significant* SwissProt hits**	3846 (53%)	2947 (52%)	3110 (48%)	3402 (55%)
# (%) with significant* trembl hits**	4172 (58%)	3134 (55%)	3353 (52%)	3645 (58%)
**# (%) with no hits**	3030 (42%)	2517 (44%)	3107 (48%)	2555 (41%)

* Reads that are submittable and > 100 bp. ** Threshold for Blastx significance = 1e-10. Gene discovery is defined as the number of different "genes" each library contributed, divided by library size. Gene Diversity is defined as the number of singletons in each library divided by library size [22].

**Table 2:** General statistics for the subtractive libraries.

	**sbs01**	**sbs02**	**sbs03**	**sbs04**
# Reads*	1021	1189	1117	1203
**# average read length (bp)**	453	485	493	482
**# singletons**	421	541	429	198
**# clusters**	155	264	194	157
**# putative transcripts**	576	805	623	355
**Avg cluster size**	3.87	2.46	3.55	6.4
**Largest cluster**	81	15	59	63
**# clusters with 2 ESTs**	113	210	146	62
**# clusters with 3 ESTs**	19	29	17	25
**# clusters with 4–5 ESTs**	10	20	16	22
**# clusters with 6–10 ESTs**	6	2	7	22
**# clusters with > 10 ESTs**	7	3	8	26
**Gene discovery**	0.56	0.68	0.56	0.30
**Gene diversity**	0.41	0.46	0.38	0.16
# (%) with significant* SwissProt hits**	308 (53%)	415 (52%)	296 (48%)	100 (28%)
# (%) with significant* trembl hits**	328 (57%)	439 (55%)	309 (50%)	110 (31%)
**# (%) with no hits**	242 (42%)	363 (45%)	309 (50%)	243 (68%)

* Reads that are submittable and > 100 bp. ** Threshold for Blastx significance = 1e-10. Gene discovery is defined as the number of different "genes" each library contributed, divided by library size. Gene Diversity is defined as the number of singletons in each library divided by library size [22].

In spite of the subtractive process, this library was still highly redundant, potentially indicating massive gene redundancy within mictic females and their associated resting eggs. Ten contigs comprise 27.4% of the library sequences. Three of the ten contigs (7.7% of the library) show no match to known sequences in the database and four contigs (12.9%) have a highly repeated amino acid structure, which cannot be ascribed to a particular gene or gene family (Table 3). None of these highly repeated proteins show similar homologies to each other, being 36–40% identical at the nucleotide level. One contig (clone sbs04P0012K21) shows similarity to putative oxidoreductases. This classification is applied to all enzymes that have oxidoreductase functions, however some are involved in acting on superoxide radicals, which are produced during stressful situations (c.f. resting eggs). Also of significant interest are the two other matches to ferritin (pearl oyster) and hsp26 (*Artemia urmiana*). In studies on the crustacean *Artemia franciscana*, which forms cysts in response to adverse conditions, two proteins were shown to be present in large amounts in the cysts; hsp26 and artemin (a ferritin homologue) [23,24]. This situation is clearly mirrored in the sbs04 library and these transcripts represent potential markers for resting eggs.

**Table 3:** Ten largest contigs in the sbs04 library (mictic females with resting eggs versus mixed stage population of clone 1B4) and the associated BLAST matches.

**Signature clone**	**No of clones**	**% of library**	**Uniprot ID**	**Description**	**e value**
sbs04P0006D10	63	5.2		Highly repeated protein	

sbs04P0012K23	44	3.6		No match	

sbs04P0011N24	40	3.3		Highly repeated protein	

sbs04P0012K21	30	2.5	Q27ST7	Putative oxidoreductase, *Hartmannella veriformis *(Amoeba)	1.1 E-49

sbs04P0012B19	28	2.3		No match	

sbs04P0012J17	28	2.3	Q7YW83	Ferritin, *Pinctada fucata *(Pearl oyster)	7.1 E-25

sbs04P0011G20	27	2.2		Highly repeated protein	

sbs04P0006P13	26	2.2		Highly repeated protein	

sbs04P0011N19	24	2.0	Q000T2	Hsp26, *Artemia urmiana *(brine shrimp)	3.6 E-7

sbs04P0011H09	22	1.8		No match	

**Total**	**332**	**27.4**			

One clone from each contig is given for EST database identification purposes.

When similarity searches were run on the processed sequences from all the libraries, approximately 50% produced significant matches (expect score in excess of 1e-10 and therefore can be regarded as putative known genes) against the sequence databases. This percentage identification is much lower than a recently published EST library of *B. plicatilis *[21], in which 80% of sequences showed similarity to database entries.

However, the number of ESTs in the Suga library was relatively small (2,362 ESTs), non-normalised and a significant proportion of the sequences formed a single cluster encoding the small ribosomal sub-unit. In total, almost 23% of the 2,362 ESTs were comprised of 14 clusters with matches ranging from cathepsin L to beta tubulin. Comparison of our ESTs with those of Suga et al., [21] using BLASTN (E value < 10^-10^) showed that 93% of the ESTs in the Suga library were represented in our dataset.

The main objective of this EST project was to develop transcriptome resources for *B. plicatilis*, which could be used in future global expression experiments. Therefore, the strategy of normalization and subtraction was used in library production. Although this should maximize the number of different transcripts obtained, it does mean that quantitative comparisons between libraries is not possible without further verification. Given this limitation, analyses were targeted at candidate genes involved in maintaining the stability and the integrity of cell compartments and macromolecules, as these are key factors for survival during dormancy. Searches were carried out using both BLAST and GO annotations and identified genes designated as or involved in:

• Protection against reactive oxygen species (ROS) and detoxification: ROS are toxic in all life stages but they are especially problematic for dormant forms. In plant seeds desiccation causes loss of control mechanisms that maintain low ROS concentrations, thus the antioxidant activity has great importance [25].

• Maintaining the native folded conformation of proteins: changes in osmotic pressure, pH or temperature as well as desiccation all challenge protein conformation [26] and may cause the formation of cytotoxic protein aggregates.

• Late Embryogenesis abundant (LEA) proteins: which have been shown to be involved in desiccation in a number of organisms [27].

• Trehalose biosynthesis: trehalose is well-known to be present in high concentrations in the dormant stages of various organisms [17] and small amounts have been found previously in *B. plicatilis *desiccated resting eggs [28].

• Aquaporins: these are transmembrane proteins that serve as channels for water and small soluble molecules transport [29] and have been found to be important for desiccation tolerance in seeds [30] and for freeze tolerance in yeast [31].

• Lipids and fatty acid metabolism: lipid metabolism is associated with hibernation in mammals [32] and the dauer form in nematodes [33]. Vitellogenins are lipoproteins forming the yolk proteins [34,35].

### Protection against ROS and detoxification

A number of clones were identified associated with antioxidant activity GO term (GO:0016209), which was specifically narrowed to encompass clones encoding glutathione S transferases (Table 4). These genes belong to a superfamily of multifunctional proteins with fundamental roles in cellular detoxification, participating in the second phase detoxification and removal of xenobiotics after the action of P450 [36]. They are widespread among all organisms. In total, 129 putative transcripts for glutathione S-transferase (E value between 9.0 e^-17 ^– 2.0 e^-45^) were found in all the normalized libraries and in the sbs04 library (seven contigs and five singletons). More in-depth analysis revealed that these 129 transcripts comprise 11 distinct putative genes (designated *Bpa-gst-1 *to *Bpa-gst-11*, where Bpa stands for *Brachionus plicatilis Atlit*), which on sequence similarity searching appear to most closely match the alpha class of cytosolic GSTs. This is by far the most abundant of cytosolic subfamilies often comprising tens of members in each species [36] (c.f. 44 annotated GSTs identified in *C. elegans *[37]). Five of the putative rotifer GSTs show closest sequence matches to *C. elegans *genes, all of which are heavily documented in Wormbase with regard to expression and functional studies. Whilst those GSTs most similar to the rotifer transcripts all show expression responses to electrophilic stress [38], interestingly GST-5 occurred in an expression cluster of strongly regulated dauer genes (WBPaper00024393; [39]). Although functions of genes (even orthologues) differ between species, and this is particularly the case with multiple gene family members, the dauer is a stage of larval arrest in *C. elegans*, which could equate functionally to the resting-egg stage in the rotifer. Indeed, this gene was only found in the resting-egg library, clearly a candidate for further investigation. No GSTs were found in the first three subtractive libraries, but this may not be surprising given the small sample size of the sequencing effort, or alternatively, their number was small as a consequence of subtractions.

**Table 4:** Putative transcripts for members of the Glutathione-S-transferase family Identified in the EST libraries.

**Contig**	**Signature clone**	**Acession number/Best BLAST match**	**Organism**	**E-value**	**MS**	**RE**	**REH**	**FRE**	**sbs04**
bpa-gst-1	sb101P0003M09	O18598/Glutathione-S-transferase	*Blattella germanica*	3 E-23	8	0	0	6	0

bpa-gst-2	sb102P0001B13	P04904/Glutathione-S-transferase alpha-3	*Rattus norvegicus*	3 E-21	5	2	6	7	0

bpa-gst-3	sb102P0027M10	Q7REH6/Glutathione-S-transferase	*Plasmodium yoelii yoelii*	5 E-24	0	1	0	1	0

bpa-gst-4	sb103P0011A24	P26697/Glutathione-S-transferase-3	*Gallus gallus*	6 E-26	0	0	6	2	0

bpa-gst-5	sb104P0024A03	Q9NAW7/Glutathione-S-transferase	*Haemonchus contortus*	5 E-33	5	0	6	13	0

bpa-gst-6	sb104P0018P05	P91253/Probable Glutathione-S-transferase-7	*Caenorhabditis elegans*	5 E-25	0	2	0	2	0

bpa-gst-7	sb102P0010B06	Q21355/Glutathione-S-transferase-4	*Caenorhabditis elegans*	9 E-23	0	11	0	4	0

bpa-gst-8	sbs04P0006G07	P91252/Probable Glutathione-S-transferase-6	*Caenorhabditis elegans*	5 E-20	0	13	0	3	11

bpa-gst-9	sb101P0040I16	P30568/Glutathione-S-transferase-A	*Pleuronectes platessa*	2 E-45	0	4	2	6	0

bpa-gst-10	sb104P0023E22	P91253/Probable Glutathione-S-transferase-7	*Caenorhabditis elegans*	5 E-28	1	0	0	1	0

bpa-gst-11	sb102P0019M19	Q09596/Probable Glutathione-S-transferase-5	*Caenorhabditis elegans*	9 E-17	0	1	0	0	0

				**Total**	**19**	**34**	**20**	**45**	**11**

Signature clones are given for transcripts (consensus sequences) composed of contigs.

Further searches for antioxidant enzymes identified 135 clones, which assembled into 11 putative transcripts coding for peroxiredoxins (E values between 10^-27 ^to 10^-77^) and thioredoxin peroxidase activity (E value of 10^-57^) (data not shown). Members of these families were found in all normalized libraries and *Bpa-trpx-6 *and *Bpa-trpx-7 *were additionally found in the sbs04 library associated with resting eggs (data not shown). Antioxidant activity is also associated with the enzyme phospholipid-hydroperoxide glutathione peroxidase, which protects membranes from oxidative stress by reducing the membrane hydroperoxides [40]. Twenty-nine clones were found to be associated with phospholipid-hydroperoxide glutathione peroxidase activity (GO:0047066) in the EST libraries and two putative transcripts were produced after contig assembly and were named *gpx1 *and *gpx2*. The transcript *gpx1 *was only found in the MS and sbs01 libraries, whilst *gpx2 *was found in all the normalized libraries. BLAST results for the two transcripts were quite different: *gpx1 *matched mammalian glutathione peroxidase 3 (E value = 10^-42^) and *gpx2 *matched phosphlipid-hydroperoxide glutathione peroxidase of hydra and cattle tick (E value = 10^-20^), and of mammals (E value 10^-18^), although both confer antioxidant protection. The presence of two genes indicates a duplication of the *gpx *genes in the rotifer.

Dismutases catalyze the conversion of superoxide radicals into hydrogen peroxide, preventing their conversion into the more active hydroxyl radical [25]. Five putative transcripts were found to be associated with superoxide dismutase activity (GO:0004784). Two transcripts show homology with the Mn-SOD (E value = 10^-111^) previously described by [19]. Three other transcripts were found to be similar to Cu/Zn-SOD. Transcripts were found across several different libraries and so could be designated as ubiquitous. However, the previously identified Mn-SOD of *B. plicatilis *was found to be over-expressed in rotifers with an extended life span resulting from caloric restriction [19]. Similarly in *C. elegans*, the DAF pathway (insulin, dauer associated) is also linked to caloric restriction and increased lifespan. Therefore these genes clearly have other roles in addition to putative functions associated with desiccation.

### Maintaining the native folded conformation of proteins

Changes in environmental conditions (e.g. osmotic pressure, pH, temperature and desiccation), challenge protein structure and may cause the formation of cytotoxic protein aggregates and induce the production of "stress" proteins [41]. Therefore, desiccation tolerant resting eggs need to develop mechanisms for coping with denaturing and aggregation of proteins. The classical cellular response to this type of stress is the induction of "heat shock" or chaperone proteins [42-44] which facilitate the disaggregation of proteins and their refolding to native conformation, and/or the production of small heat shock proteins, which prevent initial protein aggregation [26].

BLAST searches revealed 10 putative transcripts (6 contigs and 4 singletons) with matches to the HSP70 superfamily. Further analysis narrowed this to 6 putative genes as four of the sequences were potentially non-overlapping sections of the same genes (Table 5). Of the six putative genes, three showed significant sequence similarity to the classical stress inducible HSP70 gene (Bpa-hsp70-1, Bpa-hsp70-3 and Bpa-hsp-6). The best database match to this gene was from the organism *Microplitis mediator*, an orthopteran parasite and interestingly the publication annotation associated with this entry [Swiss-Prot:A8D4R0] indicates that this gene is associated with diapause. All other putative genes are HSP70 family members and although the functional annotation is variable, all are potentially involved in the stress response. HSPA9 (Bpa-hsp70-4) is additionally implicated in the control of cell proliferation and cellular aging, whilst GRP170 (Bpa-hsp70-5) has a pivotal role in cytoprotection, specifically triggered in response to hypoxia [45], both factors which are almost certainly associated with resting-egg formation. None of the rotifer sequences showed any significant similarity to the rotifer HSP70 sequence previously isolated [DDBJ:AB076052]), which is most similar to the constitutive form of this family (HSC70) and has been shown to be expressed during population growth [18]. Members of the HSP70 family were found in all normalized libraries and in two of the subtracted libraries.

**Table 5:** Putative transcripts for members of the HSP70 family identified in the EST Libraries.

**Contig**	**Signature clone**	**Organism**	**Accession number/Gene description**	**E value**	**MS**	**RE**	**REH**	**FRE**	**sbs01**	**sbs03**
bpa-hsp70-1	sb102P0043E07	*Macrobrachium rosenbergii*	Q6S4R6/Heat shock protein 70	6E-61	0	3	0	2	0	0

bpa-hsp70-2	sb103P0045K15	*Crassostrea gigas*	Q75W49/78kDa glucose regulated protein	< 1E-200	3	4	2	3	0	0

bpa-hsp70-3	sb103P0048K15	*Homo sapiens*	Q2TAL4/Heat shock 70 kDa protein 4	2E-65	2	(1)	1 (2)	1	1	0

bpa-hsp70-4	sbs01P0007O18	*Homo sapiens*	Q8N1C8/HSPA9 protein	< 1E-200	7	2	0	4 (1)	1	1

bpa-hsp70-5	sbs01P0006A07	*Rattus norvegicus*	Q6P136/Hyou1 protein (alias GRP170)	6E-37	3	0	0	0	1	0

bpa-hsp70-6	sb103P0021G10	*Microplitis mediator*	A8D4R0/Heat shock protein 70	8E-99	0	(1)	1 (2)	(1)	0	0

				**Total**	**15**	**11**	**8**	**12**	**3**	**1**

Signature clones are given for transcripts (consensus sequences) composed of contigs.

Although members of the HSP70 family are regarded as the classical cellular stress response, the small heat shock proteins are being increasingly identified as having a pivotal role in survival in stressful conditions and metabolic arrest [23]. Encysted embryos of *Artemia franciscana *have been shown to contain substantial amounts of HSP26 [46,47] along with a ferritin homologue [24], with both molecules acting as chaperones to prevent protein aggregation.

A search for small heat-shock proteins revealed five putative transcripts (5 contigs). One primarily matched an α-crystallin protein, (*Ornithodoros parkeri*, E value = 7·10^-13^), but this is not surprising as the α-crystalline domains are characteristic of small heat shock proteins [26] and indeed all the deduced amino acid sequences of putative rotifer small HSPs described here contain an α-crystallin conserved domain. This first transcript was found exclusively in the normalized libraries containing resting eggs (RE and FRE). Four additional different transcripts were identified in the subtractive sbs04 library (Table 6). Overall, small HSP transcripts were highly represented in the sbs04 library comprising 55 clones out of 1203 (~4.5%), and significantly, this was the only subtracted library to contain resting eggs. The sequence similarity of the putative rotifer small HSPs to small HSPs in the databases was low, in the region of 30% identity, but the small HSPs, contrary to the situation with HSP70, are not highly conserved between species. For example, comparing sequences from *C. briggsae *[Swiss-Prot:A8XDE7] to *C. elegans *[Swiss-Prot:P02513] and the pink hibiscus mealy bug [Swiss-Prot:A2I3W3] produces 28.6% amino acid identity/46% amino acid similarity and 29.2% identity/42.6% similarity, respectively. Given this lack of conservation, and that BLAST matches of the rotifer sequences were exclusive to other small HSPs, it is reasonable to assume that putative genes coding for small heat shock proteins are found in rotifers, particularly in resting eggs.

**Table 6:** Putative transcripts for members of the small heat shock family identified in the EST libraries.

**Gene**	**Clone**	**Accession number/Best BLAST match**	**Organism**	**E-value**	**RE**	**FRE**	**sbs02**	**sbs04**
bpa-shsp-1	sb104P0004B19	A6N9U9/Alpha crystallin	*Ornithodoros parkeri *Soft tick	7.0 E-13	8	5	0	0

bpa-shsp-2	sbs04P0011I16	Q000T2/Small heat shock protein	*Trichinella pseudospiralis *Nematode	6.0 E-7	0	0	3	13

bpa-shsp-3	sbs04P0012K07	Q000T3/Small heat shock protein	*Trichinella spiralis *Trichina worm	2.0 E-7	0	0	0	25

bpa-shsp-4	sbs04P0012F21	P02516/Heat shock protein 23	*Drosophila melanogaster *Fruit fly	6.0 E-7	0	0	0	12

bpa-shsp-5	sbs04P0012E13	P27777/16.9kDa class I heat shock protein (HSP11)	*Oryza sativa subs. Japonica *Rice	8.0 E-6	0	0	0	5

				**Total**	**8**	**5**	**3**	**55**

Signature clones are given for transcripts (consensus sequences) composed of contigs.

Regarding additional candidates for further investigation in resting-egg stage gene expression, a number of other heat shock proteins were identified (HSP60 and HSP80-100). Induction of the HSP60 protein was previously shown in *B. plicatilis *in response to various environmental pollutants [20,48], and also in *Plationus patulus *in response to arsenic and heavy metal exposure [49] and therefore are potential "stress" proteins. Eleven putative transcripts with matches to HSP60 were found, as were putative transcripts with matches to other high molecular weight heat-shock proteins (HSP80-100). These candidates were found in all of the normalized libraries.

### Late Embryogenesis Abundant (LEA) proteins

LEA proteins were originally identified in plant seeds during the late stages of embryonic development and are associated with desiccation tolerance throughout the life cycle of all major plant taxa [50]. They comprise a protein family with three major groups (Groups 1–3). They have also been found in non-plant species and to date almost all non-plant LEA proteins belong to Group 3 [51]. LEAs have been found in the nematode *Aphelenchus avenae *[52], bdelliod rotifers [53,54] and desiccated *A. franciscana *cysts [55]. The exact function of LEA proteins is as yet, unknown, but their importance in desiccation and stress tolerance has been comprehensively demonstrated. For example, silencing of the *lea *gene in *C. elegans *dauer juveniles caused a significant reduction of worm survival during induction of desiccation and in osmotic and heat stresses [56]. LEA proteins were found to prevent protein aggregation *in vitro *[57]. Also, *in vivo *experiments using *Aphelenchus avenae *LEA proteins introduced into human cell lines demonstrated that these proteins played a role in anti-aggregation and protein stabilisation during desiccation procedures [58].

**Figure 2:**
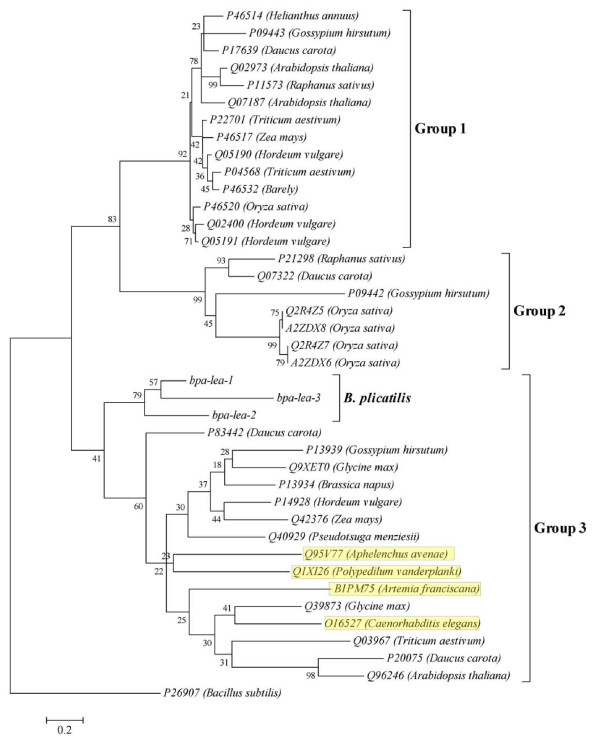
**Rooted NJ tree of *lea*-like deduced proteins, LEA proteins of other invertebrates and canonical plant LEA proteins from the three major groups**. The out-group used was of glucose starvation inducible protein of *Bacillus subtilis *(Accession No. 26907; defined as LEA protein by [51]). The canonical plant LEA proteins were chosen after [59]. The LEA proteins of invertebrates are highlighted in yellow.

Three transcripts matching group 3 LEA proteins on BLAST sequence similarity analyses were identified (E values in the range of 1E^-11 ^– 5E^-25^). These have been designated *bpa-lea-1, bpa-lea-2 *and *bpa-lea-3 *(Table 7). A rooted NJ tree was produced using translations of these transcripts with canonical plant LEA proteins from all three major groups [59] and the metazoan LEA proteins of *C. elegans, A. franciscana, P. vanderplanki*, and *A. avenae *(Figure 2). The putative rotifer genes were associated with the Group 3 protein family.

**Table 7:** Putative transcripts for Late Embryonic Abundant proteins (LEA) identified in the EST libraries.

**Contig**	**EST singnature**	**Contig size**	**Best BLAST match**	**E value**	**MS**	**RE**	**REH**	**FRE**	**sbs04**	**sbs02**
bpa-lea-1	sb104P0049I13	25	Q6NMC2/LEA-like	5E-25	0	7	1	5	12	0

bpa-lea-2	sbs04P0011H05	35	Q9FKV7/LEA-like	1E-22	3	5	3	4	20	0

bpa-lea-3	sbs02P0007H12	3	Q9FKV7/LEA-like	1E-11	0	0	0	0	2	1

				**Total**	**3**	**12**	**4**	**9**	**34**	**1**

All match transcripts from *Arabidopsis thaliana*.

### Trehalose metabolism

Trehalose is thought to play an important role in enhancing desiccation and stress tolerance [60]. For example, accumulation of trehalose has been shown in diapausing cysts of *Artemia *[61] and also the stress responses of nematodes [62,63]. Trehalose is synthesized from glucose, catalyzed by the enzymes trehalose-6-phosphate synthase (*tps*) and trehalose phosphatase [64]. Trehalose can comprise ~17% of the dry mass in *Artemia *undergoing desiccation [65] and small amounts (0.35% of dry weight) have previously been found in *B. plicatilis *desiccated resting eggs [28]. Also a transcript [DDBJ: BJ979617] with high sequence similarity to the *tps *gene, encoding to trehalose phosphate synthase, was previously identified in an EST library of *B. plicatilis *[21].

Ten ESTs (1 contig and 7 singletons) were identified in the different libraries for trehalose-6-phosphate synthase but there was no particular association with the libraries containing resting eggs. In-depth analysis revealed that the ten ESTs could be assigned to three groups comprising non-overlapping regions of the *tps *gene. In spite of this fragmentation, it was possible to identify that two paralogues (Table 8) were present and that the rotifer, like *C. elegans*, has a duplication of the *tps *gene [62]. Other model organisms, such as the insects *Drosophila melanogaster*, *Aedes aegypti*, *Anopheles gambiae *and baker's yeast, *S. cerevisiae *[66] possess only a single *tps *gene, but this may be a reflection on lifestyle and the requirement to survive stressful conditions. In support of this, phylogenetic analysis has shown adaptive selection operating on the glucose-6-phosphate branch point enzymes and adjacent pathways (including *tps*) with the conclusion that this evolutionary pressure has played a significant role in metabolic adaptation [67]. The *C. elegans *paralogues showed only 48% identity overall, but they were slightly different lengths (1229 amino acids [Swiss-Prot:O45380] (F19H8.1) and 1331 amino acids [Swiss-Prot:Q7YZT6](ZK54.2)) and particularly differed at the 5' and 3' ends. The two fragments of *tps *from the rotifer were 88.9% identical at the amino acid level, but these fragments did include the most conserved central portion of the gene and therefore the overall figure for amino acid conservation will be much lower if the whole sequence of each gene is compared.

**Table 8:** Putative transcripts for members of the trehalose-6-phosphate synthase (tps) family identified in the EST libraries.

**Contig**	**EST signature**	**Contig size**	**Best BLAST match**	**Organism**	**E value**	**MS**	**RE**	**REH**	**FRE**	**sbs01**	**sbs02**
bpa-tps-1	sb103P0045H11	6	A5XCK7/TPS	*Drosophila simulans*	1E-100	1	0	2	4	0	0

bpa-tps-2	sbs01P0007D11	3	A8D372/TPS	*Locusta migratoria manilensis*	4E-61	0	0	0	0	1	2

					**Total**	**1**	**0**	**2**	**4**	**1**	**2**

Signature clones are given for transcripts (concensus sequences).

Given the data and the nature of the way the libraries were produced it is not possible to determine the role of trehalose in resting-egg formation and survival solely using this data. In addition to the duplication of the trehalose-6-phosphate synthase gene in *C. elegans*, this species also shows a duplication of the trehalase gene, the enzyme which breaks down trehalose. In fact, there are four trehalase genes annotated in Ensembl [W05E10.4, F57B10.7, T05A12.2 and C23H3.7] [66]. BLAST searches of the rotifer data produced three singletons with matches to trehalase (data not shown). Although these were single reads and therefore sequence quality was variable, there were sufficient differences between the putative translations of these clones to indicate that they were potentially three different genes, demonstrating another situation analogous with the nematode. Although the *C. elegans *sequences are similar at the sequence level to other characterized trehalases (hence the annotation), they are designated as "unknown function", as RNAi studies produce no obvious phenotype. It has yet to be determined why there are four copies of this gene in *C. elegans *and what is the exact function of each paralogue. By extrapolation the same can be inferred for the three putative trehalases in the rotifer.

### Aquaporins

Aquaprorins are transmembrane proteins that serve as channels for water and small soluble molecules transport [29]. These proteins have been found to play a role in desiccation tolerance in seeds [30] and freeze tolerance in yeast [31]. Three different putative aquaporin transcripts were identified in the EST libraries (Table 9) with E values in the range of 6E^-22 ^– 1E^-26^. These were designated: *bpa-aqp-1*, *bpa-aqp-2 *and *bpa-aqp-3*. Exact assignment of these putative rotifer genes to aquaporin family members was difficult because of relatively short sequence lengths and low percentage similarity to aquaporin genes already in the databases. However, on BLAST assignment, the first two transcripts matched aquaporins 3, 7, 9 or 10, which are glycerol channels, while the third matched aquaporins 4, 2, 1 or the plant protein TIP. These genes are under further investigation and full length transcripts are being generated by RACE PCR for functional analyses.

**Table 9:** Putative transcripts for members of the aquaporin (aqp) family identified in the EST libraries.

Contig	EST singnature	Contig size	Accession number/Best BLAST match	Organism	E value	MS	RE	REH	FRE
bpa-aqp-1	sb104P0045O03	2	Q9YH65/Aquaporin-3	*Xenopus laevis *African clawed toad	6E-22	0	0	2	1

bpa-aqp-2	sb101P0008M24	2	A0JPL5/Aquaporin 3	*Rattus norvegicus *Rat	1E-26	2	0	0	0

bpa-aqp-3	sb102P0025K08	1	Q6T6Z9/Aquaporin-2	*Rattus norvegicus *Rat	1E-23	0	1	0	0

					Total	2	1	2	1

Signature clones are given for transcripts (concensus sequences) composed of contigs.

### Lipid and fatty acid metabolism

Also of interest were genes associated with lipid metabolism as this may be the only source for energy whilst embryonic development is arrested and during hatching if similarities are assumed with other dormant or hibernating organisms. For example lipid metabolic pathways were up-regulated in the *C. elegans *dauer larval stage [33]. Lipids also serve as the main energy source in hibernating mammals [32]. Resting eggs contain extremely large numbers of droplets with neutral lipids [68] and these may serve as the only source for biosynthetic processes during dormancy and hatching via the glyoxylate cycle and gluconeogenesis. There were 28 clones (4 contigs and 2 singletons) matching lipoprotein lipase (Table 10) in the libraries. Lipoprotein lipases are also known to serve as yolk proteins in dipterans eggs [69], in contrast to vitellogenins that are the main yolk proteins in almost all egg forming organisms [34,70]. Surprisingly, no BLAST matches were identified for vitellogenin, suggesting that lipoprotein lipase may serve as a yolk protein of *B. plicatilis*. Allied to the possession of lipoprotein lipases are fatty acid-binding proteins (FABP) which are assumed to be involved in fatty acid uptake, transport and metabolism. These proteins are members of the lipocalin superfamily that are transporters of small hydrophobic molecules such as lipids, steroid hormones, bilins and retinoids [71]. Both fatty acid and retinoid binding may be important for resting-egg formation as fatty acids may serve as an energy source during dormancy and retinoids are associated with embryonic development [72,73]. Five putative transcripts were identified as lipocalins (Table 11). For each transcript, the highest number of clones within the normalized libraries was found in library FRE (females with resting eggs) and one transcript was also found in library sbs04. These results may suggest a role of lipocalins in resting-egg production.

**Table 10:** Putative transcripts for members of the lipoprotein lipase family (lpl), members identified in the EST libraries.

**Contig**	**EST signature**	**Contig size**	**Accession number/Best BLAST match**	**Organism**	**E value**	**MS**	**RE**	**REH**	**FRE**	**sbs01**
bpa-lpl-1	sb101P0009I07	12	Q9VX01/CG6847-PA	*Drosophila melanogaster *Fruit fly	1E-37	4	0	0	8	0

bpa-lpl-2	sb104P0049B13	8	Q16LG0/Triacylglycerol lipase, pancreatic	*Aedes aegypti *Yellow fever mosquito	1E-33	3	0	0	5	0

bpa-lpl-3	sb104P0004K06	2	Q66KX1/MGC85357 protein	*Xenopus laevis *African clawed toad	3E-26	1	0	0	1	0

bpa-lpl-4	sb104P0019A08	4	Q66KX1/MGC85357 protein	*Xenopus laevis *African clawed toad	1E-43	1	0	0	1	2

bpa-lpl-5	sbs01P0007D23	1	Q16LG0/Triacylglycerol lipase, pancreatic	*Aedes aegypti *Yellow fever mosquito	1E-16	0	0	0		1

bpa-lpl-6	sb104P0004K06	1	A0MBZ6/Pancreatic lipase	*Meleagris gallopavo *Common turkey	2E-25	0	0	0	1	0

					**Total**	**9**	**0**	**0**	**16**	**3**

Signature clones are given for transcripts (concensus sequences) composed of contigs.

**Table 11:** Putative transcripts for members of the fatty acid binding proteins (fab) family identified in the EST libraries.

**Contig**	**EST singnature**	**Contig size**	**Acession number/Best BLAST match**	**Organism**	**E value**	**MS**	**RE**	**REH**	**FRE**	**sbs01**	**sbs02**	**sbs04**
bpa-fab-1	sb101P0004F07	5	Q5EBJ0/Fatty acid binding protein 3	*Mus musculus *Mouse	1E-23	1	0	1	3	0	0	0

bpa-fab-2	sb101P0022L08	10	Q90W92/Heart-type fatty acid-binding protein	*Fundulus heteroclitus *Killifish	1E-25	1	1	1	5	1	0	1

bpa-fab-3	sb104P0003D03	3	Q5EBJ0/Fatty acid binding protein 3	*Mus musculus *Mouse	6E-25	0	0	0	1	0	2	0

bpa-fab-4	sb102P0013H20	1	A8HG12/Brain-type fatty acid binding protein	*Epinephelus coioides *Orange spotted grouper	6E-18	0	1	0	0	0	0	0

bpa-fab-5	sb103P0024C16	9	Q5EBJ0/Fatty acid binding protein 3	*Mus musculus *Mouse	8E-30	0	0	2	7	0	0	0

					**Total**	**2**	**2**	**4**	**16**	**1**	**2**	**1**

Signature clones are given for transcripts (concensus sequences) composed of contigs.

### Expression experiments

**Figure 3:**
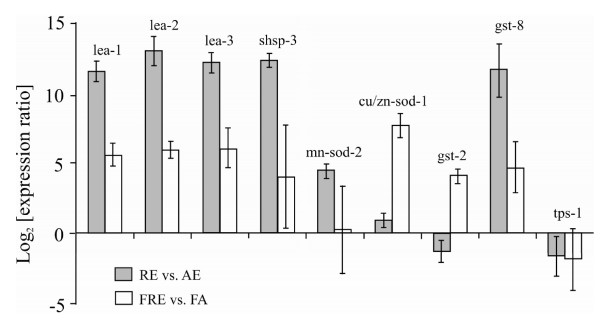
**Expression pattern of selected genes in resting eggs (RE) vs. amictic eggs (AE) and resting-egg producing females (FRE) vs. amictic females (FA)**. Genes that were tested include: the Late embryonic abundant protein (*lea-1, lea-2, lea-3*), small heat shock proteins (*shsp-3*), manganase supreroxide dismutase (*mn-sod-2*), copper or zinc superoxide dismutase (*cu/zn-sod-1*), glutathione S-trasferase (*gst-2*, *gst-8*) and trehalose phosphate synthase (*tps-1*).

Since all libraries were produced using either normalized or subtractive methods, real-time PCR experiments were conducted in order to assess the expression of selected genes in resting eggs and in resting-egg producing females (see [additional file 1] Table S1). The expression patterns of the selected genes were determined in resting eggs relative to amictic eggs, and in resting-egg producing females relative to amictic females (Fig. 3). It should be noted that in all cases the 95% confidence limits in the female samples were expanded compared to those of the egg samples. This may be attributed to the larger inherent variability between females, related to their age and size.

Genes upregulated in resting eggs include all the *lea*-like transcripts, a small heat shock protein and two of the genes involved in antioxidant activities: one of the glutathione S-transferases (*Bpa-gst-8*) and a superoxide dismutase (*Mn-sod-2*). Two *gst*-like transcripts were chosen for analysis: *Bpa*-*gst-8*, identified in the normalized libraries associated with resting eggs (RE, FRE) and also the subtracted library containing resting eggs, and *gst-2 *found in all the normalized libraries. As mentioned above, *gst-8 *is up-regulated in resting eggs and in resting-egg producing females. No significant change in the expression of *gst-2 *was found in resting eggs relative to amictic eggs but it was slightly up-regulated in resting-egg producing females. Therefore, the two gene family members clearly play different roles in cellular defense mechanisms.

The relative expression of *tps-1 *transcript was determined in order to evaluate the significance of trehalose synthesis in resting-egg production. The results do not show any significant change in the expression of the *tps-1 *like gene in resting eggs relative to amictic eggs or in resting-egg producing females relative to amictic females. Hence, the expression pattern of the *tps*-like transcript suggests that this gene may not be associated with resting-egg production, although it cannot be discounted that trehalose production is regulated at the translational level or enzyme activity rather than the transcriptional level.

## Conclusion

The production of both normalized and subtractive EST libraries from different samples of rotifer including resting-egg producing females, resting eggs and resting eggs during hatching, resulted in a high coverage of the transcriptome of *Brachionus plicatilis*. A total of 47,926 clones were sequenced, and these were assembled into 18,000 putative transcripts. Genes known to be associated with desiccation tolerance in other organisms were identified in the EST libraries. These included genes associated with antioxidant activity, low molecular weight heat shock proteins and LEA proteins. Real-time PCR confirmed that LEA transcripts, small HSPs and some antioxidant genes were upregulated in resting eggs, therefore suggesting that desiccation tolerance is a characteristic feature of resting eggs even though they do not necessarily fully desiccate during dormancy. Production of trehalose is commonly associated with dormancy and genes associated with trehalose synthesis were found in all the normalized libraries. However, the role of trehalose in resting-egg formation and survival remains unclear since there was no significant difference between resting-egg producing females and amictic females in the expression of *tps-1 *gene. Matches to lipoprotein lipase proteins suggest that, similar to the situation in dipterans, these proteins may serve as the yolk protein in rotifers and probably not vitellogenin, that is found in most other egg producing organisms. The 18,000 *Brachionus plicatilis *putative transcripts will serve as a database for future global expression experiments, particularly for the further identification of dormancy related genes.

## Methods

### Rotifer cultures and sample collection

Rotifers were hatched from resting eggs produced in the laboratory from rotifers collected at a seaside pond in Atlit, (40 km south of Haifa, Israel) in 1981. Some of the resting eggs were hatched in 2003 and resting eggs produced from them were stored in the laboratory. Resting eggs from 1981 and 2003 were stored in the dark at 4°C and hatched in 2005. Four groups of samples were collected for libraries; (I) mixed stage cultures containing both amictic and mictic females, (II) Resting eggs (RE), (III) resting eggs during hatching and (IV) mictic females with resting eggs

#### (I) Mixed stage rotifer cultures

Four cloned cultures and one non-cloned culture were grown in 400 ml sea water medium (40‰). The rotifers were fed with the algae *Nannochloropsis sp*. Mixis was induced by transferring the cultures to diluted sea water medium (20‰). Samples were collected when males appeared in the cultures. Samples for RNA extraction were collected by sieving the upper part of the culture with a plankton net (60 μm mesh). Rotifers were washed with sterile 20‰ diluted sea water and were re-suspended for 1 hr in sterile 20‰ diluted sea water, in order to allow rotifers to empty their gut content. Rotifers were sieved again with the plankton net, washed with sterile diluted sea water (20‰) and transferred into a 1.5 ml centrifuge tube. The rotifers were concentrated using a by short centrifugation step. The pellet containing the rotifers was frozen in liquid nitrogen and kept at -70°C until required for RNA extraction.

#### (II) resting egg collection

About 30,000 resting eggs were collected from two 400 ml of a non-cloned cultures that were maintained in 10‰ diluted sea water.

#### (III) resting eggs during hatching

Resting eggs were stored for three months in the dark at 25°C. Hatching was initiated by exposing the resting eggs to light. Samples were collected 20 and 30 hrs after the initiation of hatching initiation and used for the construction of the cDNA library.

#### (IV) Mictic females with resting eggs

Females with resting eggs were hand picked from cultures, due to their low abundance in the mixed cultures. About 1,000 females with resting eggs were picked from a cloned culture (clone 1B_4_) grown in a 400 ml 20‰ diluted sea water medium. This culture was also used for the production of the subtractive libraries.

### RNA extraction and library preparation

RNA was extracted with the TRIzol^® ^reagent (Invitrogen) following the manufacturer instructions. cDNA was synthesized using the SMART approach (SMART PCR cDNA synthesis kit, Clontech, U.S.A.) and subsequently normalized using duplex-specific nuclease (Trimmer kit, Evrogen, Russia) according to manufacturer's instructions and directionally cloned into pAL32 (Evrogen, Russia). Subtractive cDNAs were constructed by suppression subtractive hybridization (Evrogen, Russia) and cloned via the TOPO TA^® ^cloning system (Invitrogen, U.S.A.). Plasmids were transferred via electroporation into *E. coli *DH10B (Invitrogen, U.S.A.). Plasmids from the normalized libraries were 5' end sequenced using the pALforward primer (5'-CTCGGGAAGCGCGCCATT-3') and Big Dye Terminator chemistry. Clones of the subtractive libraries were sequenced from both ends using T7 and T3 primers.

Sequences were determined on ABI 3730XL capillary sequencers (Applied Biosystems, USA).

### cDNA libraries construction and characterisation

Four normalized libraries and four subtractive libraries were constructed:

**MS: **Normalized library of a mixed population consisting of amictic females, mictic females and males. The library was generated from a combination of four cloned cultures and one non-cloned culture

**RE: **Normalized library of resting eggs. The resting eggs were obtained from a non-cloned culture.

**REH: **Normalized library of resting eggs in various stages of hatching. The library was constructed from resting eggs that were hatched for 20 or 30 hrs.

**FRE: **Normalized library of mictic females with resting eggs. The females were collected from a resting-egg producing clone (clone 1B_4_).

**sbs01: **Subtractive library of a mixed stage population from a combination of cloned and non-cloned cultures (tester) vs. mixed stage population of a clone 1B_4 _(driver)

**sbs02: **Subtractive library of a mixes stage population of clone 1B_4 _(tester) vs. a mixed stage population of a combination of cloned and non-cloned cultures (driver).

**sbs03: **Subtractive library of a mixed stage population of clone 1B_4 _(tester) vs. mictic females with resting eggs of clone 1B_4 _(driver).

**sbs04: **Subtractive library of mictic females with resting eggs as tester vs. mixed stage population of clone 1B_4 _(driver).

### Sequence analysis and EST clustering

Sequence fasta files were processed using the script Trace2dbest [74], which incorporated the phred and crossmatch programmes [75,76]. A minimum cut-off value of 100bp was applied after quality control processing for generating the submission file for EMBL (Accession numbers, FM897377–FM945301). Tgicl [77] was used for clustering the fasta files, incorporating quality scores, for each of the five libraries, as well as for all the libraries together. The clusters were database searched using Blastx [78] against the Uniprot/Swissprot and Uniprot/Trembl databases [79], with matches annotated for all scores with an expect score in excess of 1e-10. These annotations were then used to map Gene Ontology identifiers [80]. Sequence manipulation was carried out using the EMBOSS suite of programs [81]. Multiple sequence alignments of LEA proteins were performed using the ClustalW program. [82]. Phylogenetic trees were drawn with MEGA4 [83] using the Bacillus subtilis glucose inducible starvation protein B (Accession no: P26907) as an outgroup.

### Real-Time PCR experiments

The relative abundance of *bpa-lea-1, bpa-lea-2, bpa-lea-3, bpa-shsp-3, bpa-mnsod-2, bpa-cusod-1, bpa-gst-11, bpa-gst-4, bpa-tps-1 *transcripts (see [additional file 1] Table S1 for the list of primers) were normalized to an elongation factor 1α housekeeping sequence using the equation: ratio = (E_target_)^CP_target^/(E_*ef1a*_)^CP_*ef1a *^where E = 10^-1/slop^, according to the method described by Pfaffl et al., [84]. The PCR mixture consisted of 1 μl of cDNA sample, 70 nM of each primer and 12.5 μl of SYBR Green master mix (ABgene, UK), in a final volume of 25 μl. Amplification were preformed with biological triplicate samples using a GenAmp 5700 thermocycler (PE Applied Biosystems, USA) according to the manufacturer's protocol.

## Availability

ESTs were deposited at EMBL with the accession numbers: FM897377–FM945301.

## Authors' contributions

NYD performed and led the physiological rotifer experiments, real-time PCR, bioinformatics analyses and drafted the manuscript. MAST and MSC led the data analyses and contributed to the writing of the manuscript. MK and RR were responsible of the cDNA libraries, performed the sequencing of the clones and contributed to the writing of the manuscript. EL conceived and coordinated the study, participated in the design of the experiments, analyses of data and was in charge of writing the final version of the manuscript. All authors read and approved the final manuscript.

## Supplementary Material

Additional file 1 **Table 1S: Genes and primers used in real-time PCR.** A list of primers that were used for real-time PCR experiments. Transcripts names, genes names, and primer sequences are given. Click here for file
