# Supplementary material for: Discovering genes associated with dormancy in the monogonont rotifer Brachionus plicatilis
Source: BMC Genomics. 2009 Mar 13;10:108. doi: 10.1186/1471-2164-10-108 (PMC2667189; doi:10.1186/1471-2164-10-108)
Supplement: Additional file 1 — Table 1S: Genes and primers used in real-time PCR. A list of primers that were used for real-time PCR experiments. Transcripts names, genes names, and primer sequences are given. [file 1471-2164-10-108-S1.pdf]

Table S1: List of genes and primers used in real-time PCR experiments.

Transcript name	Gene name	Forward primer	Reverse primer
bpa-ef1a	Elongation factor 1 alpha	GGTGTGGGTGAATTCGAAG	CGAATGGAATTTGTGCTGG
bpa-lea-1	Late embryogenesis abundant protein	GGTGATACCTTGGTGGTGCT	TCGCTTTTTCTTTTGCACCT
bpa-lea-2	Late embryogenesis abundant protein	AAGGAAAAGGCCGGAGAATA	TTGGCTCCCTCCACATACTC
bpa-lea-3	Late embryogenesis abundant protein	AGAACGCATGAAGGGGTCG	GCCTCAGCATAGCCAGACAA
bpa-prld-2	Mitochondrial protein	CAATCGATCCGAATAATCCAA	CCAAAATCCACTGCAAACCT
bpa-prld-3	Mitochondrial protein	GGTCTAGTTTAGGAATGAGTT	ATATACCGAAATGGGCC
bpa-shsp-3	Small heat shock protein	ACTCCGCGATCAATCAAAAC	CCTTCAGCTTCGACAACCTT
bpa-mn-sod-2	Mn superoxide dismutase	TCCGCGCCAAGCATTCTCTTCC	CAAGGCTGATGTTAAAGCTATTTGGTCG
bpa-cu/zn-sod-1	Cn/zn superoxide dismutase	CCCGGCCTATAATTCCCAAT	AAGACCCGGCAACAAAATGT
bpa-gst-2	Glutathione-S-transferase	TGTGAATCCGACAAATCCGTA	CCATGAAGTCTGAGGGAAAGC
bpa-gst-8	Glutathione-S-transferase	GTTATCTTGCACGCATTTTC	CACTTCTGGCCACAATATCT
bpa-tps-1	Trehalose-6-phosphate synthase	TCCGCACTCTTCCTCCAGAC	GTTCCCATTCAGCCCCATC
